# CellSwarm-AD: A Multi-Scale Agent-Based Framework for Virtual Alzheimer’s Disease Trials

**DOI:** 10.3390/ijms27156915

**Published:** 2026-08-01

**Authors:** Chun-Lin Tan, Xiang-Mei Zheng, Yun-Fei Zhu, Quan-Quan Xiong, Han Li, Xuan-Lin Meng

**Affiliations:** 1 State Key Laboratory of Southwestern Chinese Medicine Resources, Innovative Institute of Chinese Medicine and Pharmacy, Chengdu University of Traditional Chinese Medicine, Chengdu 611137, China; 2 Department of Biochemistry and Molecular Biology, College of Basic Medical Sciences, Naval Medical University, Shanghai 200433, China

**Keywords:** Alzheimer’s disease, agent-based modeling, virtual clinical trial, pharmacokinetics/pharmacodynamics, spatial diffusion, reproducibility

## Abstract

Computational models of Alzheimer’s disease (AD) rarely connect cellular heterogeneity, spatial tissue organization, pathology cascades, and pharmacological intervention within one auditable workflow. We present CellSwarm-AD, a four-layer framework comprising five cell agent classes, a spatial Aβ diffusion environment, an Aβ–Ca^2+^–tau–NF-κB–viability cascade with repository PK/PD models, and an optional experiment orchestration interface. Layer 3 was demonstrated with reproducible prompt templates and deterministic mock outputs; no live large language model was used to generate or modify the quantitative simulation outputs or statistical results. In a prespecified 78-week virtual trial (*n* = 200 per arm), patient-level repeated measurements were analyzed with Gaussian generalized estimating equations. Week-78 mean (SEM) MMSE-like changes were −1.747 (0.071) for the placebo, −1.368 (0.076) for lecanemab, −1.428 (0.067) for donepezil, and −1.369 (0.065) for independently simulated donepezil plus memantine. The corresponding single-trial Cohen’s d values versus the placebo were 0.395, 0.354, and 0.426. Across 20 independent *n* = 200-per-arm trials, the mean d values were 0.337, 0.359, and 0.326, respectively; replicates were not pooled. Ablation removed most of the treatment contrast when PK/PD was disabled, and fixed-domain grid testing showed decreasing relative L2 error from 6.20% (100 × 100) to 1.49% (200 × 200) against a 400 × 400 reference. These results establish a reproducible proof-of-concept while identifying calibration dependence, weak cross-layer coupling, and the absence of individual-level external validation as current limitations.

## 1. Introduction

Alzheimer’s disease (AD) is the leading cause of dementia worldwide, affecting over 55 million people and imposing an escalating burden on healthcare systems and caregivers [1]. The disease is characterized by a complex, multifactorial pathobiology in which amyloid-β (Aβ) aggregation, tau hyperphosphorylation, neuroinflammation mediated by activated microglia and reactive astrocytes, calcium dysregulation, and progressive neuronal loss interact across molecular, cellular, and tissue scales [2,3]. Despite decades of intensive research and hundreds of clinical trials, the failure rate for AD therapeutics exceeds 99% [4,5]. Recent approvals of anti-amyloid antibodies such as lecanemab have renewed optimism, yet their modest clinical effect sizes underscore the need for a deeper mechanistic understanding of how molecular interventions propagate through cellular networks to produce patient-level outcomes [6]. Computational models offer a principled route to dissect these interacting mechanisms, generate quantitative predictions, and prioritize therapeutic strategies before committing to costly and lengthy clinical trials [7,8]. However, the inherent multi-scale nature of AD—in which events at the protein level within individual cells give rise to tissue-level pathology that ultimately manifests as measurable cognitive decline—demands modeling approaches capable of bridging these biological scales within a unified framework.

Existing computational approaches to AD each illuminate important facets of the disease but remain fundamentally limited in scope and integration. Ordinary differential equation (ODE) models of amyloid kinetics, tau phosphorylation, or calcium signaling faithfully describe intracellular biochemistry and have yielded valuable insights into pathway dynamics, yet they neglect spatial organization, cell-type diversity, and stochastic variability across cell populations [9,10]. Agent-based models (ABMs) of microglial dynamics, neuronal networks, or immune cell interactions incorporate cellular heterogeneity and local decision making but typically lack mechanistic pathology cascades and pharmacological modules, limiting their translational utility [7]. Pharmacokinetic/pharmacodynamic (PK/PD) models used in clinical pharmacology accurately predict drug exposure–response relationships and are essential for dose selection, but they operate in isolation from the underlying disease biology and cannot capture how a drug’s mechanism of action interacts with the pathological microenvironment [11,12]. Critically, none of these frameworks provide an integrated computational path from molecular perturbation through cellular and tissue responses to clinical-trial-level endpoints such as cognitive scores, making it difficult to evaluate therapeutic hypotheses end-to-end. The absence of spatial modeling is particularly consequential: Aβ diffusion gradients, microglial chemotaxis toward plaques, oligodendrocyte vulnerability in white matter tracts, and the regional heterogeneity of neuronal damage are inherently spatial phenomena that cannot be faithfully represented by well-mixed compartmental models [7,8].

Here, we introduce CellSwarm-AD as a modular four-layer simulation architecture. Layer 0 contains five cell agent classes (neurons, microglia, astrocytes, oligodendrocytes, and endothelial cells). Layer 1 provides a spatial tissue grid for amyloid-beta diffusion and local interactions. Layer 2 contains the amyloid-beta-calcium-tau-NF-kappaB-viability cascade, repository PK/PD models, and the virtual trial engine. Layer 3 is an optional experiment orchestration interface demonstrated with code-faithful prompt templates and deterministic mock responses. The modules share an architecture and data interfaces, but the quantitative clinical trial path reported here calls the Layer 2 cascade and PK/PD modules rather than the Layer 1 grid. No live LLM was used to generate, select, modify, or interpret the reported numerical results. We therefore evaluate the spatial and clinical components separately and do not claim to have carried out fully coupled end-to-end four-layer validation.

We evaluate CellSwarm-AD with layer-specific numerical checks and an engine-driven virtual trial. The clinical analysis uses one prespecified n = 200-per-arm trial for inference and 20 separate trials only to characterize Monte Carlo variability. Clarity AD [6] informs the lecanemab calibration constant and is therefore not treated as an independent validation set. The external natural history comparison uses a published longitudinal AD cohort [13] and is presented as a contextual check rather than individual-level validation.

## 2. Results

### 2.1. Agent-Based Cell Dynamics Recapitulate AD Hallmarks

**Figure 1:**
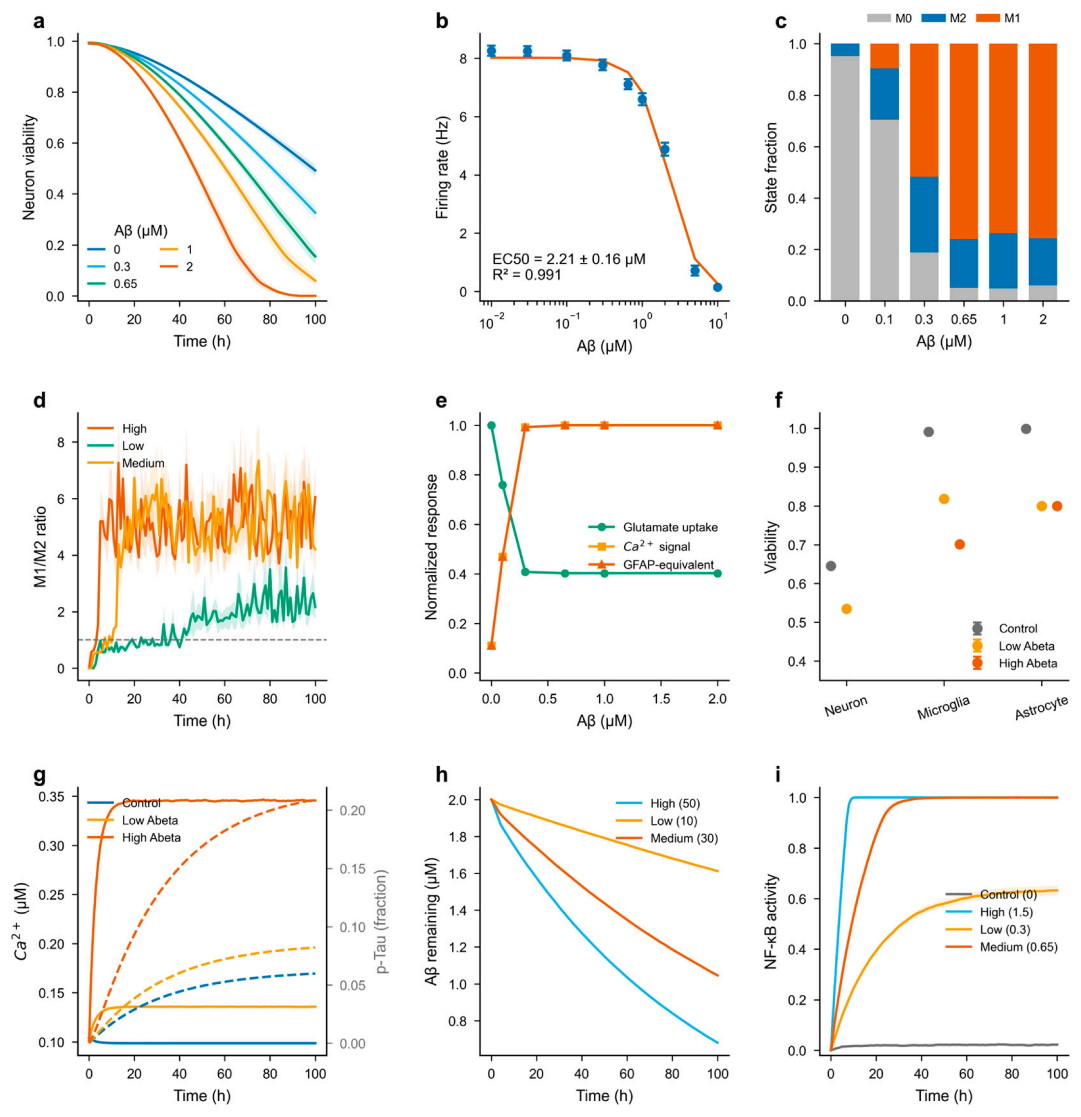
Code-derived cell agent responses under graded amyloid-beta stimulation. All summary trajectories show mean and SEM across 20 independent simulation replicates. (**a**) Neuronal viability across five amyloid-beta concentrations. (**b**) Firing rate dose response with Hill fit (EC50 = 2.21 ± 0.16 micromolar; R-squared = 0.991). (**c**) Microglial M0, M2, and M1 state fractions. (**d**) Time-dependent M1/M2 ratio under low, medium, and high amyloid-beta. (**e**) Astrocyte glutamate uptake, calcium-signal, and GFAP-equivalent responses normalized to the maximum of each series. (**f**) Mean viability and SEM for neurons, microglia, and astrocytes under control, low-amyloid-beta, and high-amyloid-beta conditions. (**g**) Neuronal calcium (solid) and p-Tau (dashed) trajectories. (**h**) Amyloid-beta remaining under three modeled microglial density conditions. (**i**) NF-kappaB activity across four amyloid-beta conditions. Lines, points, and uncertainty intervals are drawn directly from the repository processed data tables. Colors identify the Aβ dose or condition as labeled; shaded bands and error bars denote SEM. In panel (**g**), solid lines show Ca2+ and dashed lines show p-Tau, with matched colors denoting the same exposure condition.

To validate that CellSwarm-AD captures the core cellular pathology of Alzheimer’s disease, we first characterized individual cell agent responses to amyloid-β (Aβ) stimulation across a panel of concentrations (0.01–10 μM, *n* = 20 independent replicates per condition). The neuronal firing rate exhibited a sigmoidal dose–response relationship well described by a Hill function (*E*_max_ = 8.01, EC50 = 2.21 μM, Hill coefficient *n* = 2.22; Figure 1b). At the highest concentration tested (2 μM Aβ), neuronal viability declined to near zero by 100 simulated hours, whereas control neurons retained approximately 49% viability over the same interval (Figure 1a). Intracellular calcium rose to 0.346 ± 0.013 (mean ± s.e.m.) and phosphorylated tau accumulated to 0.209 ± 0.009 under high-Aβ conditions, compared with 0.099 ± 0.003 and 0.060 ± 0.002 in controls (Figure 1g). Both calcium and tau endpoints varied significantly across Aβ concentrations (one-way ANOVA: F = 287.9, *p* = 1.6 × 10^−65^ for calcium; F = 204.3, *p* = 1.0 × 10^−57^ for tau), and the two markers were tightly correlated (Pearson r = 0.98, *p* = 2.4 × 10^−63^).

Microglial agents transitioned from resting (M0) to pro-inflammatory (M1) phenotypes in a dose- and time-dependent manner [14]. Under high Aβ (1.5 μM), the M1/M2 ratio exceeded 5:1 within 5 h and remained elevated throughout the simulation (Figure 1d). Astrocyte reactivity, measured by GFAP-equivalent markers, reached full saturation at ≥0.65 μM Aβ, accompanied by the complete loss of glutamate uptake capacity (Figure 1e; Pearson r = −1.00, *p* = 2.4 × 10^−65^). NF-κB signaling showed robust dose-dependent activation (F = 2102.9, *p* = 5.2 × 10^−83^; Figure 1i), and Aβ clearance rates differed markedly across modeled microglial density conditions (F = 37,089.5, *p* = 1.7 × 10^−91^; Figure 1h). A chi-square test confirmed that the distribution of cell states across Aβ concentrations departed significantly from uniformity (χ^2^ = 2249.0, d.f. = 10, *p* < 10^−52^; Figure 1c). The 12 AD-related gene values returned by the neuron class are fixed heuristic feature weights and are not interpreted as dynamically simulated gene expression measurements.

### 2.2. Spatial Tissue Simulation Reveals Emergent Pathology Patterns

**Figure 2:**
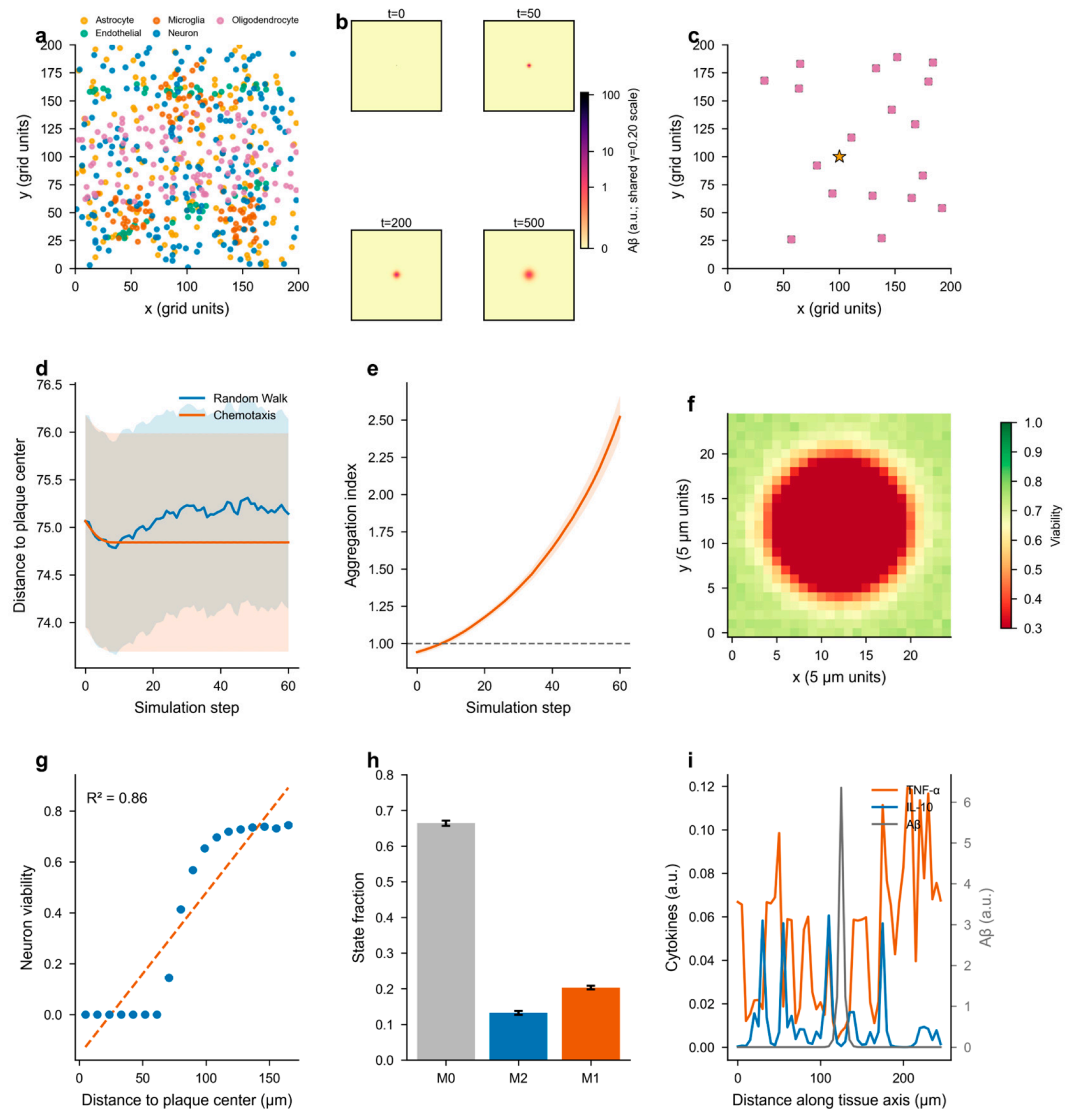
Spatial diffusion and plaque-directed cell responses in the tissue module. (**a**) Initial positions of the five modeled cell classes on a 200 × 200 grid. (**b**) Amyloid-beta field at simulation steps 0, 50, 200, and 500, shown with one shared power-normalized color scale (gamma = 0.20) to preserve both the plaque core and low-intensity spatial spread. (**c**) Representative microglial trajectories toward the plaque center (star). (**d**) Mean distance to the plaque center for random-walk and chemotaxis conditions with SEM. (**e**) Aggregation index with the random expectation (AI = 1) shown by the dashed line. (**f**) Mean neuronal viability field at the final spatial endpoint. (**g**) Binned neuronal viability versus physical distance from the plaque center (R-squared = 0.86). (**h**) Mean M0, M2, and M1 fractions across spatial replicates. (**i**) Tissue-axis profiles of TNF-alpha, IL-10, and amyloid-beta. Spatial panels and profiles use existing processed simulation outputs; no visual interpolation is used to create additional observations. In panel (**a**), blue, vermillion, yellow, pink, and green dots denote neurons, microglia, astrocytes, oligodendrocytes, and endothelial cells, respectively. In panel (**c**), gray squares and magenta circles mark trajectory starts and endpoints, and the orange star marks the plaque center. In panel (**d**), blue and vermillion lines denote random walk and chemotaxis, with shaded SEM; in panel (**i**), vermillion, blue, and gray lines denote TNF-α, IL-10, and Aβ.

We next extended the single-cell model to a two-dimensional tissue context by placing five cell types (neurons, microglia, astrocytes, oligodendrocytes, and endothelial cells) on a 200 × 200 grid with a central Aβ plaque source (Figure 2a). Aβ spread outward from the central source across simulation steps 0, 50, 200, and 500; a shared nonlinear color scale makes both the high-intensity core and lower-intensity spatial spread visible (Figure 2b). The fixed-domain grid convergence analysis in Section 2.6 provides the corresponding numerical resolution check.

The spatial model produced emergent pathology gradients: cell viability decreased monotonically with proximity to the plaque core. At the center (distance ≈ 10.9 grid units), the local Aβ concentration reached 54.9 μM and the viability was zero; beyond approximately 14 grid units (local Aβ < 0.29 μM), the viability recovered to 0.14 and continued rising to approximately 0.74 at the periphery (Figure 2f). A linear regression of viability with distance confirmed a significant positive relationship (R^2^ = 0.86, *p* = 3.2 × 10^−9^; slope = 0.032 per grid unit; Figure 2g). Microglial agents exhibited chemotaxis toward the plaque, with the mean nearest-neighbor distance converging to 74.8 grid units within approximately eight steps under directed migration, compared with random-walk controls that remained near 75.2 (Figure 2d). The spatial distribution of microglial activation states (65.5% resting, 20.5% pro-inflammatory, 13.0% anti-inflammatory across 20 replicates) did not differ significantly from the expected proportions (χ^2^ = 33.4, d.f. = 38, *p* = 0.68; Figure 2h), indicating that stochastic polarization was consistent across runs. A clustering analysis revealed that Aβ-induced damage was spatially autocorrelated, with damaged cells forming contiguous patches around the plaque (Figure 2e). Cytokine profiles showed TNF-α and IL-10 gradients radiating from the plaque center, consistent with the spatial distribution of M1 and M2 microglia (Figure 2i).

### 2.3. Pathology Cascade Exhibits Dose-Dependent Dynamics with Tau Damage as Dominant Driver

**Figure 3:**
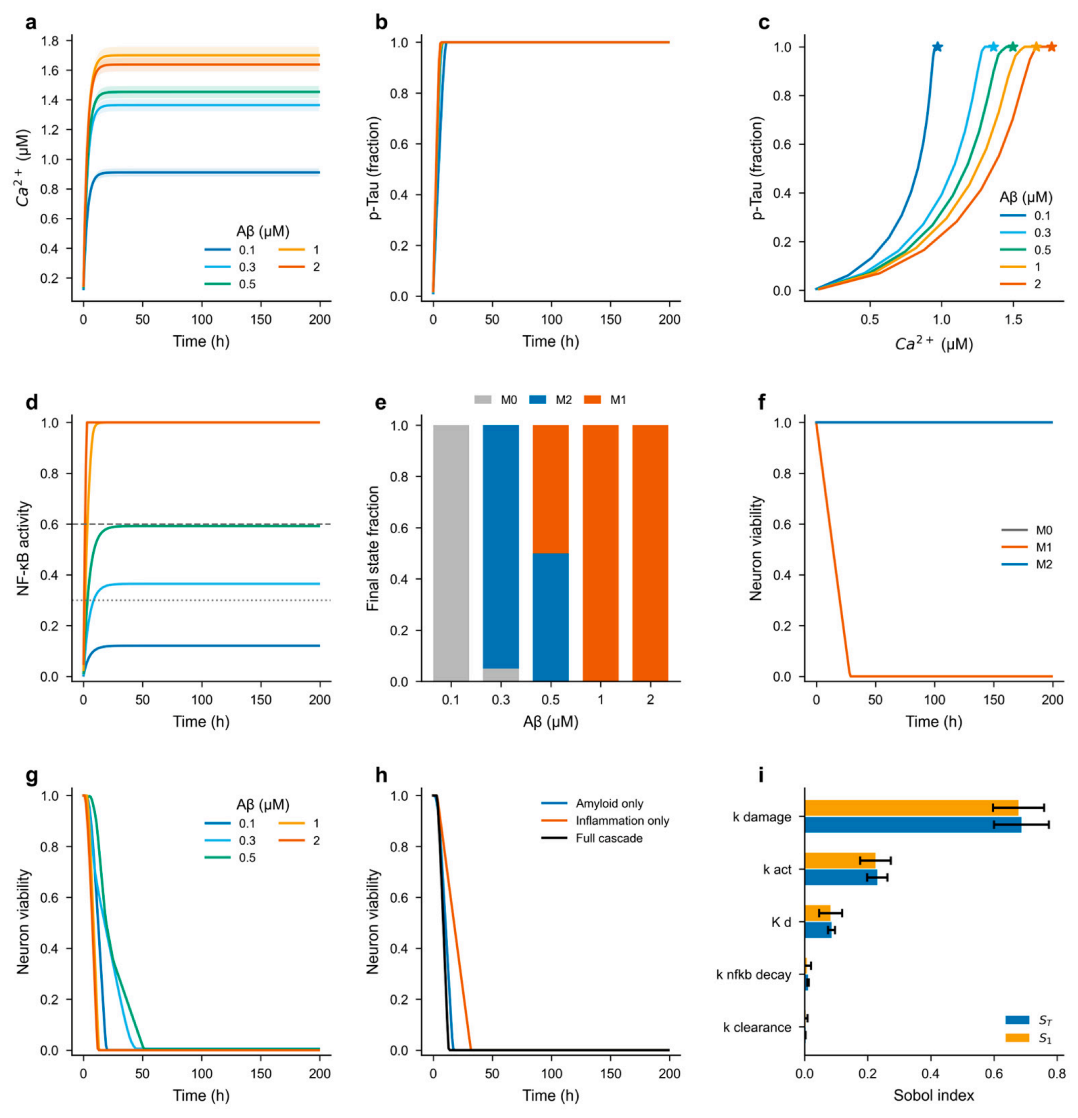
Dose-dependent pathology cascade dynamics and sensitivity. Summarized trajectories represent replicate-collapsed means from the repository processed outputs. (**a**) Intracellular calcium across five amyloid-beta inputs. (**b**) p-Tau accumulation over time for the corresponding amyloid-beta source conditions. (**c**) Mean calcium-p-Tau phase trajectories; stars mark final states. (**d**) NF-kappaB activation with M2 (0.3) and M1 (0.6) thresholds. (**e**) Final M0, M2, and M1 fractions across amyloid-beta conditions. (**f**) Neuronal viability under M0, M1, and M2 microglial states. (**g**) Full-cascade neuronal viability across amyloid-beta doses. (**h**) Amyloid-only, inflammation-only, and full-cascade decomposition at 1.0 micromolar amyloid-beta. (**i**) Total-order and first-order Sobol indices with the confidence intervals contained in the processed sensitivity table. Line colors identify Aβ dose or the state/pathway named in each legend; shaded bands and error bars denote uncertainty from the processed replicate summaries. Stars in panel (**c**) mark final states, and the dotted and dashed gray lines in panel (**d**) mark the implemented M2 (0.3) and M1 (0.6) thresholds, respectively.

To dissect the intracellular signaling cascade, we simulated the Aβ → Ca^2+^ → tau → NF-κB pathway over 200 simulated hours across five Aβ inputs (Figure 3). The Aβ-to-calcium coupling structure was informed by prior mathematical modeling of Aβ-induced intracellular calcium dysregulation [15], while the downstream tau and NF-κB links follow the literature summarized in Refs. [16,17,18,19,20]. Intracellular calcium rose rapidly to dose-dependent plateaus (Figure 3a), while phosphorylated tau accumulated toward its bounded state (Figure 3b). Calcium–p-tau phase trajectories were separated by Aβ dose (Figure 3c), and NF-κB activity crossed the implemented M2 (0.3) and M1 (0.6) thresholds as exposure increased (Figure 3d). Final microglial-state fractions consequently shifted from predominantly M0 toward M2 and M1 states with increasing Aβ (Figure 3e).

Microglial-state-specific neuronal viability trajectories are shown in Figure 3f, while the full cascade produced a dose-dependent loss of viability across Aβ inputs (Figure 3g). Decomposition at 1.0 μM Aβ showed that the full-cascade trajectory was at least as damaging as either isolated amyloid or inflammatory component (Figure 3h). Sobol global sensitivity analysis over five key rate parameters identified the tau-damage rate constant (*k*_damage_) as the dominant driver of viability outcomes, with a total-order sensitivity index *S*_T_ = 0.69 (95% CI: 0.69 ± 0.08), followed by the microglial activation rate *k*_act_ (*S*_T_ = 0.23 ± 0.03) and the calcium dissociation constant *K*_d_ (*S*_T_ = 0.09 ± 0.01; Figure 3i). NF-κB decay (*S*_T_ = 0.011) and Aβ clearance (*S*_T_ = 0.003) contributed minimally.

### 2.4. Two-Compartment PK/PD Models Predict Clinical Drug Responses

**Figure 4:**
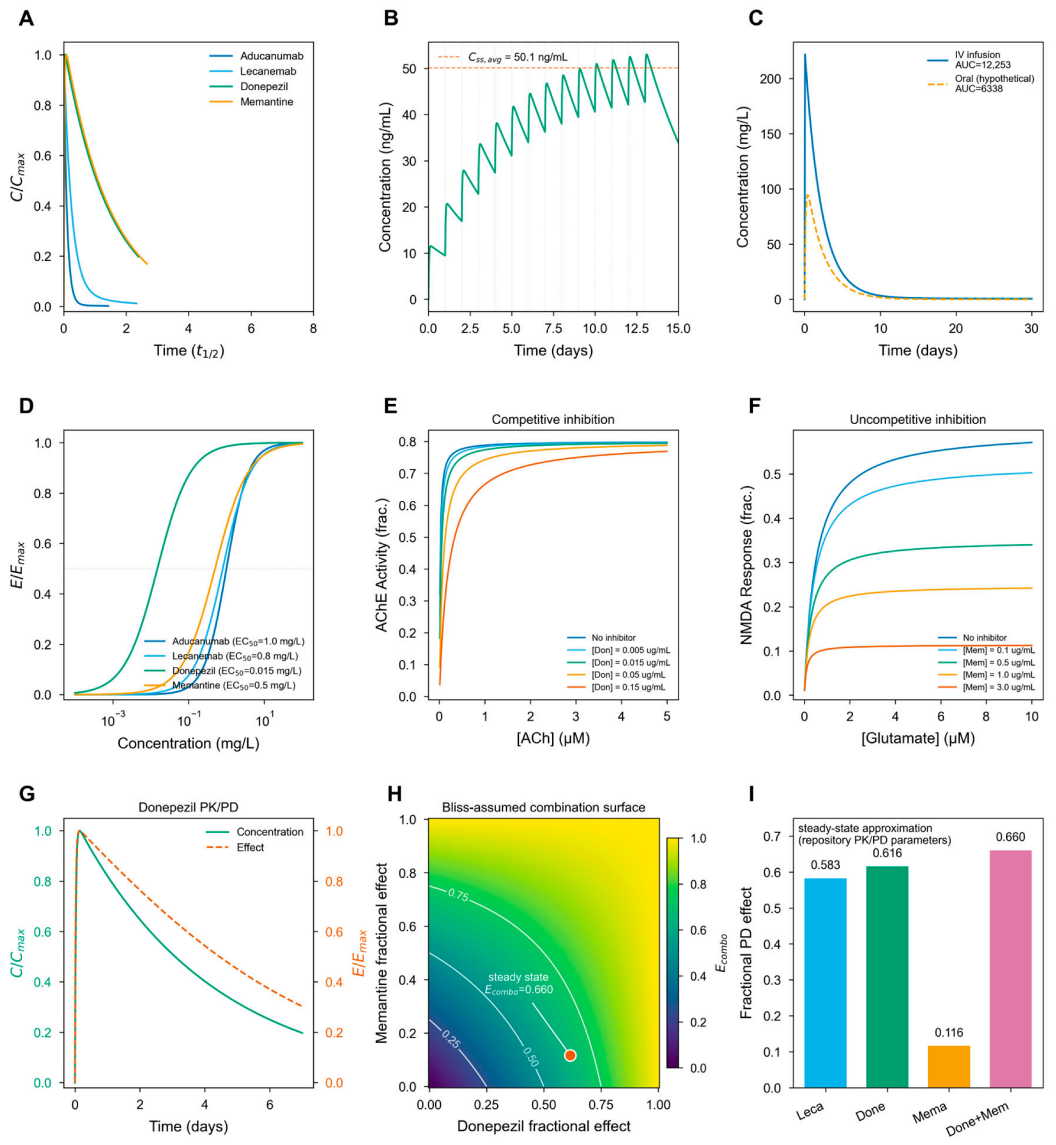
Repository PK/PD implementation and explicit combination assumption. (**A**) Normalized concentration decay for aducanumab, lecanemab, donepezil, and memantine. (**B**) Simulated lecanemab steady-state concentration. (**C**) Route-dependent exposure comparison. (**D**) Hill-type dose–response curves. (**E**) Competitive acetylcholinesterase inhibition by donepezil. (**F**) Uncompetitive NMDA-receptor inhibition by memantine. (**G**) Donepezil concentration and normalized PD effect time course. (**H**) Bliss independence response surface, Ecombo = 1 − (1 − Edonepezil)(1 − Ememantine), with contours and the repository steady-state working point (Ecombo = 0.660). The surface is used to visualize an imposed structural rule and is not an estimated drug–drug interaction. (**I**) Repository steady-state fractional PD effects for lecanemab (0.583), donepezil (0.616), memantine (0.116), and donepezil plus memantine (0.660). Panels (**H**,**I**) do not establish clinical efficacy or pharmacological synergy. In panel (**C**), solid blue and dashed orange lines denote intravenous and hypothetical oral exposure. In panel (**G**), the solid green and dashed orange lines denote normalized concentration and effect. In panel (**H**), white curves are iso-effect contours and the orange dot marks the repository steady-state working point.

We integrated pharmacokinetic and pharmacodynamic modules to simulate four AD therapeutics [21,22,23]. Anti-amyloid antibodies (aducanumab 700 mg IV, lecanemab 700 mg IV) were modeled with two-compartment PK: aducanumab achieved *C*_max_ = 221.8 μg mL^−1^ with t_1/2_ = 503.9 h, while lecanemab reached *C*_max_ = 228.0 μg mL^−1^ with t_1/2_ = 144.0 h (Figure 4A). Symptomatic agents (donepezil 10 mg oral, memantine 20 mg oral) followed one-compartment kinetics with *C*_max_ = 11.5 ng mL^−1^ (t_1/2_ = 70.0 h) and *C*_max_ = 29.9 ng mL^−1^ (t_1/2_ = 63.0 h), respectively.

Pharmacodynamic dose–response curves were represented with Hill functions for aducanumab, lecanemab, donepezil, and memantine (Figure 4D; Table 1). The mechanistic panels illustrate the implemented PK/PD behavior, including donepezil competitive inhibition, memantine uncompetitive inhibition, and modeled lecanemab amyloid-beta lowering (Figure 4E–G). For the donepezil-plus-memantine arm, Bliss independence was imposed on the common fractional PD scale as Ecombo = 1 − (1 − Edonepezil)(1 − Ememantine) (Figure 4H). This is an explicit model assumption, not an interaction estimate [24,25,26]. Repository steady-state calculations yielded fractional effects of 0.583 for lecanemab, 0.616 for donepezil, 0.116 for memantine, and 0.660 for donepezil plus memantine (Figure 4I). Clinical endpoint effects are evaluated only in the prespecified trial in Section 2.5; the earlier unsupported preliminary efficacy panel was removed.

### 2.5. Virtual Clinical Trial Outcomes and Simulation Variability

**Figure 5:**
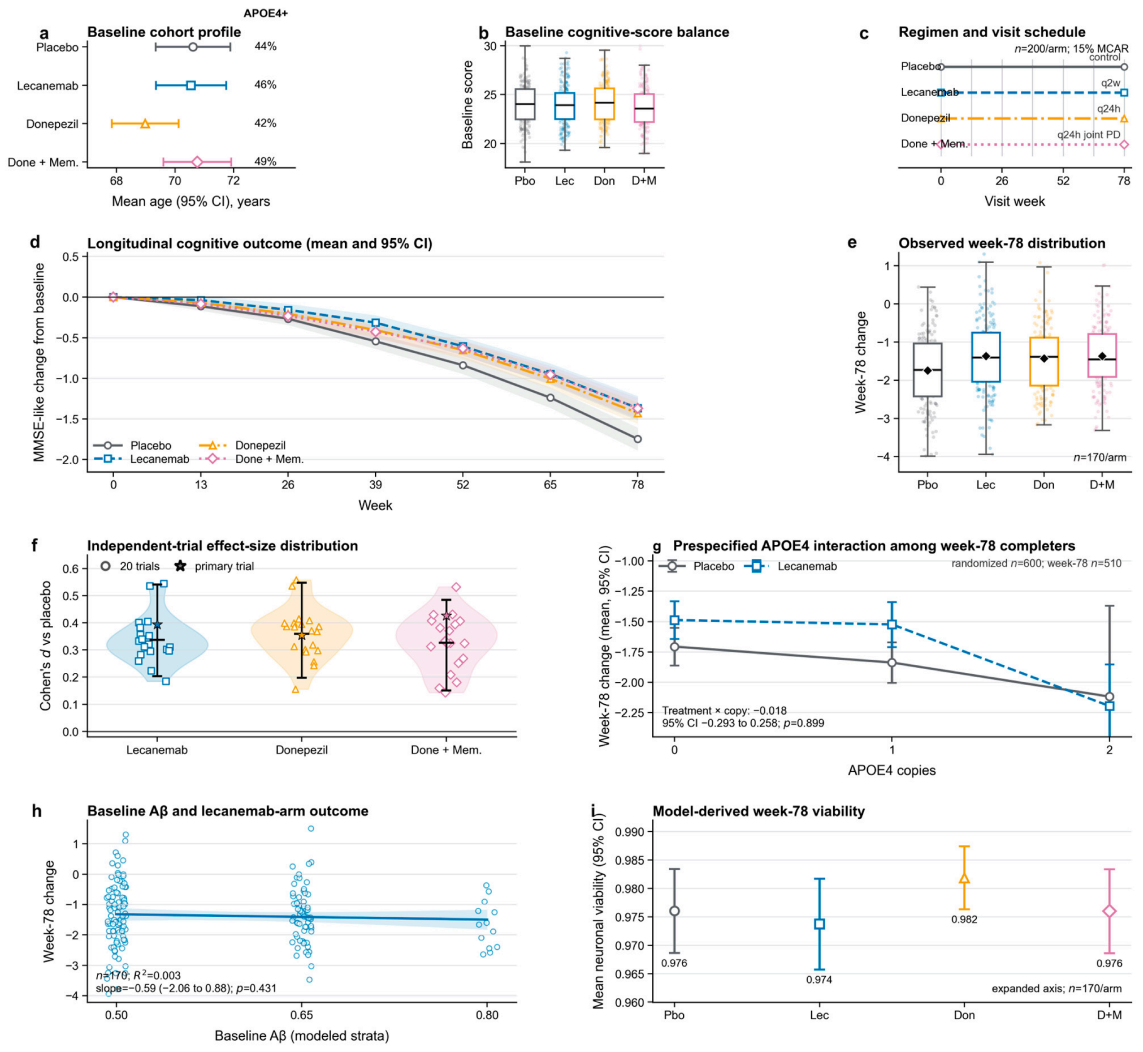
Evidence-bound nine-panel summary of the engine-driven 78-week virtual clinical trial. (**a**) Mean age with 95% confidence intervals and APOE4 carrier prevalence in each randomized arm (n = 200 per arm). (**b**) Patient-level baseline MMSE-like score distributions; points are observed virtual patients and boxes show medians and interquartile ranges. (**c**) Repository dosing intervals and the prespecified seven-visit schedule; 15% of patients were missing completely at random for monotone dropout, assigned independently of outcomes, in each arm. (**d**) Mean MMSE-like change from baseline with 95% confidence intervals across observed visits; color, marker shape, and line style redundantly identify treatment. (**e**) Patient-level week-78 distributions among observed completers (n = 170 per arm); diamonds show means and boxes show medians and interquartile ranges. (**f**) Cohen’s d values from 20 independently seeded *n* = 200-per-arm trials. Open markers are individual trial estimates, black intervals show the mean and 2.5th–97.5th percentiles, and stars identify the primary single-trial estimate; trials were not pooled. (**g**) Prespecified treatment-by-APOE4-copy analysis in a separately simulated placebo/lecanemab cohort (600 randomized; 510 week-78 completers). Completer cell sizes for 0, 1, and 2 copies were 140, 97, and 12 for placebo and 141, 105, and 15 for lecanemab. Points show completer means and 95% confidence intervals; the interaction was −0.018 MMSE-like points per copy (95% CI −0.293 to 0.258; *p* = 0.899). (**h**) Baseline amyloid-beta strata versus week-78 change among 170 observed lecanemab completers. Baseline amyloid-beta comprised three modeled strata, and horizontal jitter is display-only; the HC3 regression slope was −0.59 (95% CI −2.06 to 0.88; *p* = 0.431; R-squared = 0.003). (**i**) Descriptive mean model-derived neuronal viability with 95% confidence intervals at week 78 (n = 170 per arm); the ordinate is expanded and no between-arm inferential claim is made. Donepezil plus memantine was simulated as a distinct arm using the repository PK/PD models and Bliss independence as an explicit structural assumption, not an estimated drug–drug interaction. Across panels (**c**,**d**), gray solid circles, blue dashed squares, orange dash-dot triangles, and pink dotted diamonds identify placebo, lecanemab, donepezil, and donepezil plus memantine, respectively; shaded bands in panel (**d**) are 95% CIs. In panel (**e**), black diamonds denote means. In panel (**f**), open markers denote the 20 independent trials, stars denote the primary trial, and black intervals show the mean and 2.5th–97.5th percentiles. In panel (**g**), circles/solid gray and squares/dashed blue identify placebo and lecanemab and error bars are 95% CIs. In panel (**h**), open circles are completers, the blue line is the HC3 regression, and the blue band is its 95% CI; panel (**i**) markers and error bars denote arm means and 95% CIs.

We conducted a prespecified 78-week virtual trial with 200 patients per arm (placebo, lecanemab, donepezil, and donepezil plus memantine; 800 randomized patients). The Mini-Mental State Examination (MMSE)-like cognitive score was evaluated at weeks 0, 13, 26, 39, 52, 65, and 78. Baseline age, APOE4-carrier prevalence, and MMSE-like score distributions are shown without post-randomization screening in Figure 5a,b. The repository regimen intervals and planned outcome visits are summarized in Figure 5c. Thirty patients per arm were selected independently of outcomes for monotone dropout, yielding exactly 15% cumulative monotone missingness per arm under a missing-completely-at-random (MCAR) mechanism. All observed visits were analyzed using a patient-clustered Gaussian generalized estimating equation with categorical visit, treatment-by-visit terms, exchangeable working correlation, baseline adjustment, and robust sandwich standard errors; no imputation or last-observation-carried-forward procedure was used.

Longitudinal means and 95% confidence intervals are shown in Figure 5d. At week 78, the mean (SEM) change was −1.747 (0.071) for placebo, −1.368 (0.076) for lecanemab, −1.428 (0.067) for donepezil, and −1.369 (0.065) for donepezil plus memantine (170 observed completers per arm; Figure 5e). GEE contrasts versus placebo were 0.368 (95% CI 0.169–0.568; *p* = 0.00030) for lecanemab, 0.315 (0.128–0.503; *p* = 0.00095) for donepezil, and 0.370 (0.185–0.554; *p* = 0.000087) for donepezil plus memantine. Single-trial Cohen’s d values were 0.395 (95% CI 0.180–0.609), 0.354 (0.140–0.568), and 0.426 (0.211–0.641), respectively (Table S4).

Twenty additional independently seeded trials, each retaining *n* = 200 randomized patients per arm, quantified simulation variability rather than increasing the inferential sample size. Mean replicate-level Cohen’s d was 0.337 for lecanemab (2.5th-97.5th percentile 0.203–0.540), 0.359 for donepezil (0.197–0.547), and 0.326 for donepezil plus memantine (0.151–0.484; Figure 5f). The separately simulated APOE4 analysis included 600 randomized patients and used the 510 patients with an observed week-78 outcome for the prespecified endpoint interaction. It showed no significant treatment effect modification (interaction −0.018 MMSE-like points per allele, 95% CI −0.293 to 0.258; *p* = 0.899; Figure 5g). Within the primary lecanemab arm, the three modeled baseline amyloid-beta strata explained little week-78 outcome variation (HC3 slope −0.59, 95% CI −2.06 to 0.88; *p* = 0.431; R-squared = 0.003; Figure 5h). The mean model-derived week-78 neuronal viability was 0.976 for the placebo, 0.974 for lecanemab, 0.982 for donepezil, and 0.976 for donepezil plus memantine; these descriptive values were not subjected to a between-arm efficacy test (Figure 5i). The combination did not consistently exceed donepezil monotherapy across independent trials. Because Bliss independence was imposed on absolute PD effects rather than estimated as a clinical interaction, these simulations do not constitute evidence of pharmacological synergy.

### 2.6. Calibration, External Context, Ablation, and Numerical Convergence

The clinical scaling constant k = 0.34 was derived from the 27.1% slowing reported in Clarity AD [6] and was fixed before the present revision analyses. Clarity AD is therefore a calibration source, not an independent validation dataset. As an external contextual check, a prospective longitudinal cohort of 702 patients with AD reported an average Mini-Mental State decline of 2.15 points per year (95% CI 1.85–2.46) during the first two years [13], whereas the present placebo simulation produced −1.747 points over 78 weeks. The model therefore underestimates decline relative to that published cohort; differences in disease stage, outcome construction, and cohort composition preclude a claim of external validation. In the separate APOE4 analysis cohort, 510 of 600 randomized patients had an observed week-78 outcome. A completer-only endpoint analysis found no significant treatment-by-APOE4-copy interaction (−0.018 MMSE-like points per allele, 95% CI −0.293 to 0.258; *p* = 0.899).

The current TrialSimulator does not call the Layer 1 tissue grid, so an end-to-end clinical ablation of the spatial layer is not defined in the present implementation; patient trial ablations and the tissue spatial numerical benchmark were therefore analyzed separately. In a separate n = 300-per-group donepezil-plus-memantine versus placebo ablation experiment, the full model produced a mean MMSE-like difference of 0.395 points (95% CI 0.234–0.555). Flattening baseline Aβ heterogeneity produced 0.390 points (0.231–0.549), removing the NF-κB inflammatory state produced 0.471 points (0.304–0.638), and disabling PK/PD reduced the contrast to 0.086 points (−0.077 to 0.249). These results show that the treatment contrast depends on PK/PD but that the present inflammatory implementation does not improve predictive validity. In a separate fixed 1 mm × 1 mm diffusion benchmark, grids of 100 × 100, 200 × 200, and 400 × 400 used dx-dependent Laplacian scaling, stable timesteps, a fixed physical source, and a fixed physical region of interest. Relative L2 error versus the restricted 400 × 400 reference decreased from 6.20% to 1.49%, supporting the 200 × 200 numerical choice while retaining a measurable discretization error.

## 3. Discussion

CellSwarm-AD provides a reproducible proof-of-concept for linking a pathology cascade and repository PK/PD models to patient-level virtual trial analysis while retaining separate cell agent and tissue spatial modules. In the prespecified 78-week trial, single-trial Cohen’s d values versus placebo were 0.395 for lecanemab, 0.354 for donepezil, and 0.426 for donepezil plus memantine. Across 20 independently simulated trials, the corresponding mean replicate-level d values were 0.337, 0.359, and 0.326. The combination did not consistently exceed donepezil monotherapy and therefore provides no evidence of synergy. Because the lecanemab scaling constant was calibrated from Clarity AD [6], the trial cannot be interpreted as an independent external validation. The slower placebo decline relative to the published longitudinal AD natural history cohort [13] further indicates a residual calibration error. Accordingly, the present results support auditability and hypothesis generation rather than prospective predictions of treatment efficacy.

The main design contribution is modular rather than a claim that every layer is fully coupled in the present endpoint analysis. The cell agent classes encode cell-specific state updates, the spatial module represents diffusion and local organization, and the pathology/PK/PD module connects amyloid-beta, calcium, tau, inflammation, viability, and drug effects. For the reported clinical trial, however, TrialSimulator calls the pathology cascade and PK/PD implementations directly and does not execute the Layer 1 tissue grid. The spatial benchmark and virtual trial should therefore be interpreted as separate validations of repository components, not as an experimentally validated end-to-end tissue-to-cognition chain. Likewise, Layer 3 remains an optional software interface with a mock default backend and was excluded from the quantitative inference. This explicit boundary makes the current evidence reproducible and identifies full cross-layer coupling and prospective external validation as directions for future work.

Several limitations should be acknowledged. Clarity AD [6] was used for calibration and cannot simultaneously serve as independent validation. The published longitudinal AD cohort [13] provides only a contextual natural history comparison, and the slower simulated placebo decline indicates residual calibration error. The current virtual trial path calls the cascade and PK/PD modules but not the spatial tissue grid; cross-layer integration is therefore architectural rather than fully coupled for the trial endpoint. The donepezil-plus-memantine effect is generated under Bliss independence, which is a structural assumption rather than inferred synergy. The APOE4 endpoint analysis was restricted to simulated week-78 completers and detected no significant treatment-by-genotype interaction. Multiple parameter combinations may produce similar model outputs, and the present sensitivity and provenance analyses do not establish formal parameter identifiability. Finally, Layer 3 was demonstrated through deterministic mock responses; no live LLM was used to generate, select, modify, or interpret the reported quantitative results.

These limitations point toward productive future directions. An extension to three-dimensional tissue volumes, potentially leveraging GPU-accelerated agent simulation, would enable the modeling of cortical layer-specific vulnerability and white matter degeneration tracked by oligodendrocyte agents. Patient-specific parameterization—initializing virtual patients from cerebrospinal fluid biomarkers (Aβ42/40 ratio, p-tau181, NfL), APOE genotype, and volumetric MRI—could transform CellSwarm-AD from a population-level tool into a precision medicine platform for individual trial enrichment and outcome prediction. Joint training of cascade parameters against longitudinal clinical datasets (for example, ADNI, A4 Study) using Bayesian calibration or simulation-based inference would further constrain the model and quantify parametric uncertainty. The virtual trial engine naturally extends to combinatorial drug optimization: a systematic exploration of anti-Aβ plus anti-tau or anti-inflammatory combination regimens, dose scheduling, and treatment sequencing could identify synergistic strategies that are prohibitively expensive to test in real RCTs. Finally, tighter integration of the LLM orchestrator with the simulation engine could enable conversational hypothesis generation, automated sensitivity analysis, and natural-language reporting of trial outcomes, lowering the barrier for non-computational researchers to engage with mechanistic disease modeling.

## 4. Methods

### 4.1. Cell Agent Models

CellSwarm-AD represents brain tissue as a collection of autonomous cell agents belonging to five types: neurons, microglia, astrocytes, oligodendrocytes, and endothelial cells (blood–brain barrier units). Each agent maintains a set of continuous state variables updated at every simulation timestep *dt*.

Neurons track viability, cytosolic calcium, tau phosphorylation, and a 12-gene AD-related feature vector (APP 1.00, PSEN1 0.80, PSEN2 0.60, APOE4 0.90, TREM2 0.70, MAPT 0.85, BIN1 0.50, CLU 0.55, ABCA7 0.45, CD33 0.40, SORL1 0.65, and ADAM10 0.35). We correct the original description: these dimensionless weights are heuristic model assignments used to initialize relative features; they were not directly estimated from ROSMAP or another transcriptomic dataset. Gene selection is biologically motivated, but the numerical weights should not be interpreted as measured expression fold changes. The code and Supplementary Table S5 identify them accordingly.

Microglia are modeled with three activation states—M0 (resting), M1 (proinflammatory), and M2 (anti-inflammatory)—governed by NF-κB activity. NF-κB is activated by Aβ (rate 0.1 × [Aβ] × *dt*) and decays at 5% per unit time. State transitions are probabilistic: at NF-κB > 0.6, microglia adopt M1 with probability of 0.75 and M2 with probability of 0.20; at NF-κB 0.3–0.6, M1 and M2 probabilities are 0.30 and 0.40, respectively; below 0.1, cells remain M0 with probability of 0.95. M1 microglia produce TNF-α (0.8 × NF-κB) and IL-1β (0.6 × NF-κB) with low phagocytosis (rate ~0.1); M2 microglia produce IL-10 (0.3 × NF-κB) and TGF-β (0.2 × NF-κB) with high phagocytosis (rate ~0.6).

Astrocytes track reactivity (0–1), glutamate uptake capacity, and calcium wave amplitude. Reactivity increases with cytokine exposure and Aβ concentration; glutamate uptake decreases linearly with reactivity (from 0.8 at rest to 0.1 at full reactivity), modeling the loss of homeostatic support observed in reactive astrogliosis.

Oligodendrocytes progress through three maturation states (OPC → pre-OL → mature OL), with transitions gated by Aβ and TNF-α exposure. Myelination capacity degrades with Aβ exposure, and myelin integrity degrades with elevated calcium, reflecting white matter vulnerability in AD.

Endothelial cells maintain BBB integrity and tight junction strength, both degraded by Aβ deposition and TNF-α. Net Aβ transport rate across the BBB depends on barrier health, modeling the shift from LRP1-mediated efflux (intact BBB) to RAGEmediated influx (compromised BBB). Full code-faithful update equations for all five agent classes are provided in Table S6.

Unless a specific analysis states otherwise, parameter variability is generated only where the executable script explicitly samples a distribution. The previous blanket statement that all agent parameters used 10% CV was inaccurate and has been removed. Supplementary Table S5 distinguishes runtime defaults, sampled parameters, numerical controls, and heuristic/model-assigned coefficients. Because the available summary targets do not uniquely constrain all coefficients, these provenance and sensitivity checks should not be interpreted as a formal parameter identifiability analysis.

### 4.2. Spatial Tissue Environment

The principal spatial benchmark uses a 200 × 200 grid at 5 μm per grid unit (1 mm × 1 mm). For each of 20 figure replicates, the executable generator samples 180–219 neurons, 90–109 microglia, 140–159 astrocytes, 90–109 oligodendrocytes, and 45–54 endothelial cells. Neurons are placed uniformly; microglia are clustered around three specified centers; astrocytes are distributed across three y-axis bands; oligodendrocytes are restricted to y = 60–139; and endothelial cells are placed along two vessel-like lines. These are simulation design counts and placement rules, not estimates of absolute cortical cell density. Aβ diffusion uses the finite-difference implementation documented in the code, and grid convergence is reported in Section 2.6.

### 4.3. Pathology Cascade

The AD pathology cascade integrates two parallel signaling pathways converging on neuronal viability.

**Amyloid pathway (Aβ → Ca^2^**^+^ **→ tau → neuron death).** Aβ oligomers increase cytosolic calcium via NMDA receptor-mediated influx, modeled as Michaelis–Menten saturation kinetics:

Ca^2+^_influx_ = *k*_act_ · [Aβ]/(*K_A_* + [Aβ])

where *k*_act_ = 0.5 (maximum influx rate) and *K*_A_ = 0.1 (half-saturation constant). These dimensionless coefficients are repository defaults selected for the bounded response; Ref. [15] supports the Aβ–calcium coupling structure rather than the numerical values. Calcium clearance follows first-order kinetics toward a resting concentration (*Ca*_rest_ = 0.1 μM) with aggregate rate constant *k*_clearance_ = 0.3 s^−1^. This rate is a repository default, while Ref. [27] supports the mechanistic interpretation of ATP-dependent neuronal calcium clearance involving PMCA and SERCA. Elevated calcium activates GSK-3β and CDK5, increasing tau phosphorylation:

k_phos,eff = k_phos,base · (1 + α · max(0, [Ca^2+^] − Ca_thr))

where *k*_phos,base_ = 0.05, *α* = 2.0, and *Ca*_thr_ *=* 0.2 μM [16]. Tau dephosphorylation by PP2A proceeds at rate *k*_dephos_ = 0.03 s^−1^ [17]. Phosphorylated tau damages neurons via a Hill function:

damage = k_damage · [p-tau]^n^/(K_d^n^ + [p-tau]^n^)

with *k*_damage_ = 0.1, *K*_d_ = 0.5, and Hill coefficient *n* = 2.0 [18].

**Inflammatory pathway (Aβ → microglia → neuron).** Aβ activates microglia through TLR4 signaling, modulated by TREM2-mediated inhibition:

activation = k_act · [Aβ] · (1 − TREM2_inh)

where *k*_act_ = 0.3 and *TREM2*_inh_ = 0.2 [19]. NF-κB decays via IκBα-mediated negative feedback at rate *k*_decay_ = 0.2 s^−1^ [20]. Microglial effects on neurons are state-dependent: M1 microglia damage neurons through TNF-α (*k*_damage_ = 0.05), while M2 microglia confer partial protection through TGF-β (*k*_protect_ = 0.10) [14]. All state variables are clamped to physiological ranges (Ca^2+^: 0.05–5.0 μM; p-tau: 0–1; NF-κB: 0–1; viability: 0–1). The complete executable pathology cascade defaults are provided in Table S1.

### 4.4. Drug Pharmacokinetic and Pharmacodynamic Models

Drug disposition is modeled using compartmental pharmacokinetics [21,22,23,24,25,26]. Monoclonal antibodies (aducanumab, lecanemab) follow a two-compartment model solved numerically via scipy.integrate.solve_ivp (SciPy; https://scipy.org/; accessed on 27 July 2026) (RK45, relative tolerance 10^−9^):

dC_c/dt = R(t)/V_c − k_e·C_c − k_12_·C_c + k_21_·C_p·(V_p/V_c)

dC_p/dt = k_12_·C_c·(V_c/V_p) − k_21_·C_p

where *C*_c_ and *C*_p_ are central and peripheral compartment concentrations, *R*(*t*) is the infusion rate, and *k*_e_, *k*_12_, and *k*_21_ are micro-rate constants. Small molecules (donepezil, memantine) follow a one-compartment model with first-order absorption:

*C*(*t*) = *D*·*k*_a_·*F*/[*V*_d_(*k*_a_ − *k*_e_)] · (e^−k^_e_^·t^ − e^−kₐ·t^)

Pharmacodynamic effects are computed using the E_max_ (Hill) model:

E(C) = E_max · C^n^/(EC_50_^n^ + C^n^)

Drug-specific parameters are summarized in Table 1. The complete executable parameter set is listed in Table S2.

### 4.5. Virtual Clinical Trial Design

The authoritative trial analysis randomizes 800 generated mild-stage patients equally to placebo, lecanemab, donepezil, or donepezil plus memantine. Longitudinal outcomes are derived from the ADCascade and repository PK/PD implementations at weeks 0, 13, 26, 39, 52, 65, and 78. The combination arm is simulated independently using the two repository one-compartment PK models and Bliss independence, Ecombo = 1 − (1 − Edonepezil)(1 − Ememantine), on the common absolute fractional PD scale. This rule is an explicit structural assumption and is not an estimated interaction.

Dropout uses exact-quota missing completely at random sampling within arm: 30 of 200 patients are selected without reference to outcomes and assigned a first missing post-baseline visit, after which missingness is monotone. The cascade uses nine steps per week and dt = 0.01. At each scheduled visit, the pathology score combines viability loss (0.5), p-tau (0.3), and normalized calcium (0.2). Gaussian independent observation noise increments yield SD = 0.9 at week 78, matching the endpoint noise scale in TrialSimulator.

The calibration factor k = 0.34 was derived from the Clarity AD lecanemab slowing estimate and is therefore treated as calibrated rather than independently validated. It was held fixed for the present analyses. No donepezil, memantine, combination, APOE4 interaction, ablation, or grid convergence result was used to refit k. The source code, seed, patient-level visit table, analysis metadata, and replicate-level Monte Carlo output are included in the revision archive.

### 4.6. Sensitivity Analysis

Parameter sensitivity was assessed in two stages. First, one-at-a-time (OAT) screening varied each of 14 cascade parameters by ±20% from nominal values, recording the change in three outputs: final neuron viability, p-tau level, and cognitive decline at 78 weeks. Second, variance-based global sensitivity analysis was performed using the Sobol method implemented in SALib (https://salib.readthedocs.io/; accessed on 27 July 2026) (*N* = 512 base samples, Saltelli sampling scheme). The top five most influential parameters identified by firstorder Sobol indices (*S*_1_) were further analyzed with ±30% perturbation ranges. Totalorder indices (*S*_T_) were computed to quantify interaction effects (Supplementary Table S3).

### 4.7. Large Language Model Orchestrator

Layer 3 is an optional software interface for experiment design and narrative synthesis. ReasoningChain and ExperimentChain construct prompts from a user-supplied simulation context and pass them through LLMWrapper. The repository default and fallback backend is deterministic mock software. No live LLM was used to generate, select, modify, or interpret the quantitative results in Figure 1, Figure 2, Figure 3, Figure 4 and Figure 5. Supplementary Table S7 reproduces the executable prompt templates and fixed mock outputs and distinguishes this demonstrated behavior from potential external-backend use.

### 4.8. Statistical Analysis

All tests were two-sided with α = 0.05. The primary longitudinal analysis used a patient-clustered Gaussian GEE with treatment, categorical visit, treatment-by-visit interactions, baseline MMSE-like score, exchangeable working correlation, and Huber–White robust standard errors. Observed visits contributed under the simulated MCAR mechanism; no imputation was performed. Week-78 standardized mean differences used pooled-SD Cohen’s d with approximate 95% confidence intervals. Monte Carlo results summarize one effect estimate per independently simulated trial. The APOE4 endpoint analysis used the 510 simulated patients with observed week-78 outcomes and patient-level ordinary least squares with an APOE4-copy-by-treatment interaction and HC3 covariance. Ablation mean differences used Welch intervals. Grid convergence used relative L2, region-of-interest, and total mass errors against the restricted 400 × 400 solution. Supplementary Note S1 documents the code-to-manuscript provenance audit, and Supplementary Note S2 documents the reproducible numerical-validation workflow.

## Figures and Tables

**Table 1:** Pharmacokinetic and pharmacodynamic parameters.

Drug	Model	*V*_c_ or *V*_d_ (L)	*k*_e_ (h^−1^)	*k*_12_ (h^−1^)	*k*_21_ (h^−1^)	*t*_1/2_ (h)	*E*_max_	EC_50_ (μg/mL)	Hill *n*	Route	Dose
Aducanumab	2-comp	3.1	0.0178	0.00134	0.00149	~504	0.70	1.0	1.5	IV q4w	10 mg/kg
Lecanemab	2-comp	3.0	0.0222	0.00521	0.00625	~144	0.59	0.8	1.2	IV q2w	10 mg/kg
Donepezil	1-comp	840	0.0099	—	—	~70	0.80	0.015	1.0	PO daily	10 mg
Memantine	1-comp	630	0.011	—	—	~63	0.60	0.5	1.0	PO BID	20 mg

## Data Availability

The complete source code, deterministic seeds, patient-level simulated data, analysis scripts, and revision outputs are publicly available at https://github.com/dawnmengsjtu/CellSwarm-AD, accessed on 27 July 2026. The same curated source, data, tests, and rebuild instructions are included in the accompanying supplementary software archive. The GitHub repository was accessed on 27 July 2026.

## Supplementary Materials

The following supporting information can be downloaded at: https://www.mdpi.com/article/10.3390/ijms27156915/s1.
