# Supplementary material for: CellSwarm-AD: A Multi-Scale Agent-Based Framework for Virtual Alzheimer’s Disease Trials
Source: Int J Mol Sci. 2026 Aug 1;27(15):6915. doi: 10.3390/ijms27156915 (PMC13467131; doi:10.3390/ijms27156915)
Supplement: Supplementary file 1 [file ijms-27-06915-s001.zip › ijms-4396966-supplementary.pdf]

Supplementary Materials

CellSwarm-AD: a multi-scale agent-based framework for virtual Alzheimer's disease trials

---

Supplementary Table S1: Complete Pathology Cascade Parameters

Module 1: Aβ → Ca^{2+} Coupling (AbetaCalciumCoupling)

Parameter	Symbol	Default	Unit	Description
Aβ-Ca^{2+} activation rate	k_abeta_ca	0.5	s^{-1}	Maximum Ca^{2+} influx rate via NMDA receptors
Half-saturation constant	K_m	0.1	μM	Michaelis–Menten constant for Aβ activation
Ca^{2+} clearance rate	k_clearance	0.3	s^{-1}	PMCA/SERCA-mediated calcium clearance
Resting Ca^{2+}	ca_rest	0.1	μM	Baseline cytosolic calcium concentration

Module 2: Ca^{2+} → Tau Coupling (CalciumTauCoupling)

Parameter	Symbol	Default	Unit	Description
Base phosphorylation rate	k_phos_base	0.05	s^{-1}	Baseline tau phosphorylation by GSK-3β/CDK5
Ca^{2+} amplification factor	α	2.0	—	Calcium-dependent kinase amplification
Ca^{2+} threshold	ca_threshold	0.2	μM	Threshold for calcium-dependent kinase activation
Dephosphorylation rate	k_dephos	0.03	s^{-1}	PP2A-mediated tau dephosphorylation

Module 3: Aβ → Microglia Coupling (AbetaMicrogliaCoupling)

Parameter	Symbol	Default	Unit	Description
Microglial activation rate	k_act	0.3	s^{-1}	TLR4-mediated NF-κB activation by Aβ
TREM2 inhibition	trem2_inhibition	0.2	—	TREM2-mediated dampening of activation (0–1)
NF-κB decay rate	k_nfkb_decay	0.2	s^{-1}	IκBα-mediated negative feedback

Module 4: Tau → Neuron Damage (TauNeuronCoupling)

Parameter	Symbol	Default	Unit	Description
Maximum damage rate	k_damage	0.1	s^{-1}	Hill-function maximum viability loss rate
Half-max damage concentration	K_d	0.5	—	p-Tau concentration for 50% max damage
Hill coefficient	n	2.0	—	Cooperativity of tau-mediated damage

Module 5: Microglia → Neuron Coupling (MicrogliaNeuronCoupling)

Parameter	Symbol	Default	Unit	Description
TNF-α damage coefficient	k_damage	0.15	s^{-1}	M1 microglia neurotoxicity via TNF-α
TGF-β protection coefficient	k_protect	0.10	s^{-1}	M2 microglia neuroprotection via TGF-β

Supplementary Table S2: Drug PK/PD Parameters (Extended)

Parameter	Aducanumab	Lecanemab	Donepezil	Memantine
**PK Model**	Two-compartment	Two-compartment	One-compartment	One-compartment
Dose	700 mg (10 mg/kg)	700 mg (10 mg/kg)	10 mg	20 mg
Route	IV infusion (1h)	IV infusion (1h)	Oral	Oral
Schedule	q4w	q2w	Daily	BID
Vc or Vd (L)	3.1	3.0	840	630
Vp (L)	2.8	2.5	—	—
ke (h^{-1})	0.01780	0.02223	0.00990	0.01100
k12 (h^{-1})	0.00134	0.00521	—	—
k21 (h^{-1})	0.00149	0.00625	—	—
ka (h^{-1})	—	—	1.5	0.8
F	1.0	1.0	1.0	1.0
t½ (h)	503.9	144.0	70.0	63.0
**PD Model**	Emax (Hill)	Emax (Hill)	Emax (Hill)	Emax (Hill)
Emax	0.70	0.59	0.80	0.60
EC50 (mg/L)	1.0	0.8	0.015	0.5
Hill n	1.5	1.2	1.0	1.0
Drug type	Antagonist	Antagonist	Antagonist (competitive)	Antagonist (uncompetitive)
Ki	—	—	0.012	0.7
Year approved	2021	2023	1996	2003
Clinical reference	EMERGE/ENGAGE	CLARITY AD	Rogers 1998	Reisberg 2003

Supplementary Table S3: Sobol Global Sensitivity Analysis Results

Analysis performed on top-5 parameters identified by OAT screening (±30% perturbation, N=512 Saltelli samples, output: neuron viability at t=5 a.u., Aβ=1.0 μM).

Parameter	Description	S_1	S_1 95% CI	S_T	S_T 95% CI	Rank
k_damage	Tau→neuron damage rate	0.678	±0.083	0.687	±0.084	1
k_act	Aβ→Ca^{2+} activation rate	0.225	±0.057	0.230	±0.033	2
K_d	Damage half-saturation	0.083	±0.032	0.085	±0.011	3
k_nfkb_decay	NF-κB decay rate	0.007	±0.012	0.011	±0.002	4
k_clearance	Ca^{2+} clearance rate	0.003	±0.006	0.003	±0.000	5

S_1: first-order Sobol index (main effect). S_T: total-order index (main + interactions). Sum of S_1 = 0.996, indicating minimal higher-order interactions among top-5 parameters.

Supplementary Table S4: MMRM Full Results

Mixed-model repeated measures: cognitive_change ~ arm × week + baseline_cognitive + age + C(apoe4_copies) + (1|patient_id). Holm–Bonferroni correction applied.

Drug vs Placebo	Main effect (β)	Main p	Interaction (β/week)	Interaction p	95% CI	Total Δ @ Week 78	Adjusted p	Reject H_0
Lecanemab	−0.059	0.498	0.01275	$<10^{-300}$	[0.01218, 0.01333]	+0.936 MMSE	$<10^{-300}$	Yes
Donepezil	−0.060	0.483	0.00702	$<10^{-300}$	[0.00645, 0.00760]	+0.487 MMSE	$<10^{-300}$	Yes
Combination	−0.015	0.866	0.01372	$<10^{-300}$	[0.01314, 0.01430]	+1.056 MMSE	$<10^{-300}$	Yes

Effect sizes (Cohen's d vs Placebo): Lecanemab d=0.46 (95% CI: 0.27–0.66), Donepezil d=0.25 (95% CI: 0.05–0.44), Combination d=0.51 (95% CI: 0.31–0.71).

Supplementary Note S1: Mathematical Model Derivation

The AD pathology cascade is governed by the following system of ordinary differential equations, updated at each simulation timestep dt:

Equation S1: Calcium dynamics

$$dCa^{2+}/dt = k\_abeta\_ca \cdot [A\beta] / (K\_m + [A\beta]) - k\_clearance \cdot (Ca^{2+} - ca\_rest)$$

The first term represents NMDA receptor-mediated calcium influx following Michaelis–Menten saturation kinetics (k_abeta_ca = 0.5, K_m = 0.1 μM). The second term models active calcium

clearance by PMCA pumps and SERCA toward the resting concentration ca_rest = 0.1 μM (k_clearance = 0.3 s^{-1}). At steady state ($dCa^{2+}/dt = 0$):

Ca^{2+}_ss = ca_rest + (k_abeta_ca / k_clearance) · [Aβ] / (K_m + [Aβ])

Equation S2: Tau phosphorylation dynamics

dpTau/dt = k_phos_eff · (1 − pTau) − k_dephos · pTau

where k_phos_eff = k_phos_base · (1 + α · max(0, Ca^{2+} − ca_threshold))

Phosphorylation is calcium-dependent: when Ca^{2+} exceeds the threshold (0.2 μM), GSK-3β and CDK5 are activated with amplification factor α = 2.0. PP2A-mediated dephosphorylation proceeds at rate k_dephos = 0.03 s^{-1}. The (1 − pTau) term ensures saturation at pTau = 1.

Equation S3: NF-κB dynamics

dNFκB/dt = k_act · [Aβ] · (1 − trem2_inhibition) − k_nfkb_decay · NFκB

Aβ activates NF-κB through TLR4 signaling (k_act = 0.3), modulated by TREM2-mediated inhibition (trem2_inhibition = 0.2, reducing effective activation by 20%). IκBα-mediated negative feedback drives NF-κB decay (k_nfkb_decay = 0.2 s^{-1}).

Equation S4: Neuron viability (tau-mediated damage)

dViability/dt = −k_damage · pTau^n / (K_d^n + pTau^n)

Phosphorylated tau damages neurons via a Hill function with cooperativity n = 2.0, half-max concentration K_d = 0.5, and maximum damage rate k_damage = 0.1 s^{-1}.

Equation S5: Neuron viability (microglia-mediated)

dViability/dt += −(k_damage_mg · TNFα − k_protect · TGFβ)

where cytokine levels depend on microglial state:

- M0: TNFα = 0.1, TGFβ = 0.1 (net effect: mild damage)

- M1: TNFα = 0.8, TGFβ = 0.05 (net effect: strong damage)

- M2: TNFα = 0.05, TGFβ = 0.6 (net effect: protection)

Microglial state transitions are probabilistic, governed by NF-κB thresholds (M1 at NFκB > 0.6, M2 at 0.3–0.6, M0 at NFκB < 0.1).

Combined viability update

At each timestep, viability is updated by both tau-mediated and microglia-mediated damage:

Viability(t+dt) = clamp(Viability(t) + [Eq.4 + Eq.5] · dt, 0, 1)

Supplementary Note S2: Virtual Clinical Trial Design Details

Patient generation algorithm

Virtual patients are generated by sampling from the following distributions:

- Age: Uniform(55, 85) years

- APOE4 copies: Binomial($n=2$, $p=0.25$), yielding ~56% non-carriers, ~38% heterozygous, ~6% homozygous

- Disease stage: Configurable mixture (default: mild=0.6, moderate=0.35, severe=0.05)

- Baseline MMSE: Normal(μ_stage, σ_stage), clamped to [0, 30]

- Mild: $\mu=24.0$, $\sigma=2.0$

- Moderate: $\mu=17.0$, $\sigma=2.5$

- Severe: $\mu=8.0$, $\sigma=3.0$

- Baseline Aβ burden: 1.0 + 0.3 × APOE4_copies (APOE4 increases amyloid load by 30% per allele)

Dropout model

Cumulative dropout rate: 15% over 78 weeks. Per-step dropout probability: 0.15 / total_steps, applied uniformly at each simulation step. Patients who drop out retain their last observed cognitive score (last observation carried forward).

Cognitive decline model

MMSE change = −base_decline × (0.05 + 0.95 × pathology_score) + noise

where:

- base_decline = STAGE_DECLINE_RATE × (duration_weeks / 52) × fraction_completed

- Mild: 2.5 MMSE points/year

- Moderate: 3.2 MMSE points/year

- Severe: 5.0 MMSE points/year

- pathology_score = 0.5 × viability_loss + 0.3 × pTau_burden + 0.2 × calcium_norm

- Drug effect: pathology_score × (1 − PD_effect × 0.55)

- noise ~ Normal(0, 0.9) MMSE points

MMRM specification

Model: cognitive_change ~ arm × week + baseline_cognitive + age + C(apoe4_copies) + (1|patient_id)

Implemented using statsmodels MixedLM with REML estimation. The arm × week interaction term captures differential rates of cognitive decline between treatment groups. Multiple comparisons corrected using Holm–Bonferroni procedure across 3 drug-vs-placebo contrasts.

Effect size calculation

Cohen's d = (mean_drug − mean_placebo) / SD_pooled

where SD_pooled = sqrt((SD²_drug + SD²_placebo) / 2)

95% confidence intervals computed using the normal approximation: d ± 1.96 × SE_d, where SE_d = sqrt((n_drug + n_placebo) / (n_drug × n_placebo) + d² / (2 × (n_drug + n_placebo))).

Supplementary Table S5. Code-derived parameter provenance

Parameter	Value	Unit	Provenance class
AstrocyteAgent.agent_id	AS_0	not explicitly declared	heuristic/model-assigned
AstrocyteAgent.reactivity	0.0	code-normalized / not explicitly declared	heuristic/model-assigned
AstrocyteAgent.glutamate_uptake	0.8	not explicitly declared	heuristic/model-assigned
AstrocyteAgent.calcium_wave	0.0	code-normalized / not explicitly declared	heuristic/model-assigned
AstrocyteAgent.step.abeta_concentration	0.0	code-normalized / not explicitly declared	heuristic/model-assigned
AstrocyteAgent.step.cytokine_level	0.0	not explicitly declared	heuristic/model-assigned
AstrocyteAgent.step.dt	0.01	simulation-time unit	heuristic/model-assigned
EndothelialAgent.agent_id	EC_0	not explicitly declared	heuristic/model-assigned
EndothelialAgent.bbb_integrity	1.0	code-normalized / not explicitly declared	heuristic/model-assigned
EndothelialAgent.tight_junction_strength	1.0	code-normalized / not explicitly declared	heuristic/model-assigned
EndothelialAgent.abeta_transport_rate	0.5	code-normalized / not explicitly declared	heuristic/model-assigned
EndothelialAgent.ABETA_TJ_COEFF	0.04	code-normalized / not explicitly declared	heuristic/model-assigned
EndothelialAgent.TNF_INTEGRITY_COEFF	0.1	code-normalized / not explicitly declared	heuristic/model-assigned
EndothelialAgent.IL10_PROTECTION_COEFF	0.06	code-normalized / not explicitly declared	heuristic/model-assigned
EndothelialAgent.TJ_REPAIR_RATE	0.01	code-normalized / not explicitly declared	heuristic/model-assigned
EndothelialAgent.LRP1_BASELINE	0.6	not explicitly declared	heuristic/model-assigned
EndothelialAgent.RAGE_INFLUX_COEFF	0.3	code-normalized / not explicitly declared	heuristic/model-assigned
EndothelialAgent.step.abeta_concentration	0.0	code-normalized / not explicitly declared	heuristic/model-assigned
EndothelialAgent.step.tnf_alpha	0.0	not explicitly declared	heuristic/model-assigned
EndothelialAgent.step.il10	0.0	not explicitly declared	heuristic/model-assigned
EndothelialAgent.step.dt	0.01	simulation-time unit	heuristic/model-assigned
MicrogliaAgent.agent_id	MG_0	not explicitly declared	heuristic/model-assigned
MicrogliaAgent.nfkb_activity	0.0	code-normalized / not explicitly declared	heuristic/model-assigned
MicrogliaAgent.phagocytosis_rate	0.3	code-normalized / not explicitly declared	heuristic/model-assigned
MicrogliaAgent.step.abeta_concentration	0.0	code-normalized / not explicitly declared	heuristic/model-assigned
MicrogliaAgent.step.dt	0.01	simulation-time unit	heuristic/model-assigned
GENE_MAP.ABCA7	0.45	not explicitly declared	heuristic/model-assigned
GENE_MAP.ADAM10	0.35	not explicitly declared	heuristic/model-assigned
GENE_MAP.APOE4	0.9	not explicitly declared	heuristic/model-assigned
GENE_MAP.APP	1.0	not explicitly declared	heuristic/model-assigned
GENE_MAP.BIN1	0.5	not explicitly declared	heuristic/model-assigned
GENE_MAP.CD33	0.4	not explicitly declared	heuristic/model-assigned
GENE_MAP.CLU	0.55	not explicitly declared	heuristic/model-assigned
GENE_MAP.MAPT	0.85	not explicitly declared	heuristic/model-assigned
GENE_MAP.PSEN1	0.8	not explicitly declared	heuristic/model-assigned
GENE_MAP.PSEN2	0.6	not explicitly declared	heuristic/model-assigned
GENE_MAP.SORL1	0.65	not explicitly declared	heuristic/model-assigned
GENE_MAP.TREM2	0.7	not explicitly declared	heuristic/model-assigned
NeuronAgent.agent_id	N_0	not explicitly declared	heuristic/model-assigned

Parameter	Value	Unit	Provenance class
NeuronAgent.viability	1.0	code-normalized / not explicitly declared	heuristic/model-assigned
NeuronAgent.calcium	0.1	code-normalized / not explicitly declared	heuristic/model-assigned
NeuronAgent.tau_phosphorylation	0.0	code-normalized / not explicitly declared	heuristic/model-assigned
NeuronAgent.step.abeta_concentration	0.0	code-normalized / not explicitly declared	heuristic/model-assigned
NeuronAgent.step.dt	0.01	simulation-time unit	heuristic/model-assigned
OligodendrocyteAgent.agent_id	OL_0	not explicitly declared	heuristic/model-assigned
OligodendrocyteAgent.myelination_capacity	0.8	code-normalized / not explicitly declared	heuristic/model-assigned
OligodendrocyteAgent.myelin_integrity	1.0	code-normalized / not explicitly declared	heuristic/model-assigned
OligodendrocyteAgent._maturation_progress	0.0	code-normalized / not explicitly declared	heuristic/model-assigned
OligodendrocyteAgent.ABETA_MYELINATION_COEFF	0.05	code-normalized / not explicitly declared	heuristic/model-assigned
OligodendrocyteAgent.CALCIUM_INTEGRITY_COEFF	0.08	code-normalized / not explicitly declared	heuristic/model-assigned
OligodendrocyteAgent.TNF_MATURATION_INHIBITION	0.7	not explicitly declared	heuristic/model-assigned
OligodendrocyteAgent.BASE_MATURATION_RATE	0.02	code-normalized / not explicitly declared	heuristic/model-assigned
OligodendrocyteAgent.OPC_TO_PRE_OL_ THRESHOLD	0.4	code-normalized / not explicitly declared	heuristic/model-assigned
OligodendrocyteAgent.PRE_OL_TO_MATURE_ THRESHOLD	0.8	code-normalized / not explicitly declared	heuristic/model-assigned
OligodendrocyteAgent.step.abeta_concentration	0.0	code-normalized / not explicitly declared	heuristic/model-assigned
OligodendrocyteAgent.step.calcium_level	0.1	code-normalized / not explicitly declared	heuristic/model-assigned
OligodendrocyteAgent.step.tnf_alpha	0.0	not explicitly declared	heuristic/model-assigned
OligodendrocyteAgent.step.dt	0.01	simulation-time unit	heuristic/model-assigned
AbetaCalciumCoupling.k_abeta_ca	0.5	not explicitly declared	heuristic/model-assigned
AbetaCalciumCoupling.K_m	0.1	not explicitly declared	heuristic/model-assigned
AbetaCalciumCoupling.k_clearance	0.3	not explicitly declared	heuristic/model-assigned
AbetaCalciumCoupling.ca_rest	0.1	not explicitly declared	heuristic/model-assigned
AbetaMicrogliaCoupling.k_act	0.3	not explicitly declared	heuristic/model-assigned
AbetaMicrogliaCoupling.trem2_inhibition	0.2	not explicitly declared	heuristic/model-assigned
AbetaMicrogliaCoupling.k_nfkb_decay	0.2	code-normalized / not explicitly declared	heuristic/model-assigned
CalciumTauCoupling.k_phos_base	0.05	not explicitly declared	heuristic/model-assigned
CalciumTauCoupling.alpha	2.0	not explicitly declared	heuristic/model-assigned
CalciumTauCoupling.ca_threshold	0.2	code-normalized / not explicitly declared	heuristic/model-assigned
CalciumTauCoupling.k_dephos	0.03	not explicitly declared	heuristic/model-assigned
CascadeState.abeta_oligomer	0.0	not explicitly declared	heuristic/model-assigned
CascadeState.calcium	0.1	code-normalized / not explicitly declared	heuristic/model-assigned
CascadeState.p_tau	0.0	code-normalized / not explicitly declared	heuristic/model-assigned
CascadeState.viability	1.0	code-normalized / not explicitly declared	heuristic/model-assigned
CascadeState.nfkb	0.0	code-normalized / not explicitly declared	heuristic/model-assigned
CascadeState.microglia_state	M0	not explicitly declared	heuristic/model-assigned
CascadeState.time	0.0	not explicitly declared	heuristic/model-assigned
CascadeState.abeta_production_rate	0.1	code-normalized / not explicitly declared	heuristic/model-assigned

Parameter	Value	Unit	Provenance class
CascadeState.abeta_clearance_rate	0.02	code-normalized / not explicitly declared	heuristic/model-assigned
CascadeState.k_tau_to_abeta	0.05	code-normalized / not explicitly declared	heuristic/model-assigned
ADCascade.step.dt	0.01	simulation-time unit	heuristic/model-assigned
ADCascade.run.dt	0.01	simulation-time unit	heuristic/model-assigned
ADCascade.run.steps	100	steps	heuristic/model-assigned
ADCascade.step_with_feedback.dt	0.01	simulation-time unit	heuristic/model-assigned
ADCascade.run_with_feedback.dt	0.01	simulation-time unit	heuristic/model-assigned
ADCascade.run_with_feedback.steps	100	steps	heuristic/model-assigned
MicrogliaNeuronCoupling.k_damage	0.05	not explicitly declared	heuristic/model-assigned
MicrogliaNeuronCoupling.k_protect	0.1	not explicitly declared	heuristic/model-assigned
TauNeuronCoupling.k_damage	0.1	not explicitly declared	heuristic/model-assigned
TauNeuronCoupling.K_d	0.5	not explicitly declared	heuristic/model-assigned
TauNeuronCoupling.n	2.0	not explicitly declared	heuristic/model-assigned

The complete machine-readable table additionally records source detail, line number, code path, and declaration kind in `selected_runtime_parameters.csv`.

Supplementary Table S6. Layer 3 prompts and evidence boundary

The following strings reproduce the prompt templates present in executable source. Braced fields are run-time substitutions. The repository default and fallback backend is deterministic mock software; no archived request/response log demonstrates that a live model generated the quantitative results in the manuscript.

S6-P1 – reasoning_chain.py / HYPOTHESIS

User prompt template. `Based on the following AD simulation context, generate a testable hypothesis. Context: {context} Formulate a specific, mechanistic hypothesis:`

System prompt. `You are an Alzheimer's Disease research scientist.`

S6-P2 – reasoning_chain.py / ANALYSIS

User prompt template. `Analyze the following AD simulation results. Results: {context} Provide a detailed scientific analysis:`

System prompt. `You are an Alzheimer's Disease research scientist.`

S6-P3 – reasoning_chain.py / SYNTHESIS

User prompt template. `Synthesize the following findings into a coherent narrative. Findings: {context} Synthesis:`

System prompt. `You are an Alzheimer's Disease research scientist.`

S6-P4 – reasoning_chain.py / CRITIQUE

User prompt template. `Critically evaluate the following hypothesis and evidence. Content: {context} Critique:`

System prompt. `You are an Alzheimer's Disease research scientist.`

S6-P5 – experiment_chain.py / design_experiment

User prompt template. `Design a simulation experiment to test this hypothesis: Hypothesis: {hypothesis} Available parameters: {params_str} Specify: experiment name, parameter ranges, duration, and metrics to track.`

System prompt. `You are an AD computational experiment designer.`

S6-P6 – experiment_chain.py / refine_experiment

User prompt template. `Refine this experiment based on results: Original: {spec.name} -- {spec.description[:200]} Results: {results} Suggest refined parameters and metrics.`

System prompt. `No system prompt is supplied by this call.`

Backend boundary. The Anthropic adapter supplies a generic scientific-assistant system prompt only when its system-prompt argument is empty. The OpenAI adapter omits a system message when it is empty. The local backend posts only the prompt and maximum-token value. These adapters are software capabilities; they are not evidence that a live provider was used in the reported analyses.

Supplementary Table S7. Code-faithful Layer 0 agent updates

This reconstruction follows the executable `step()` methods. Let Δt denote the update interval, A the amyloid-β input, and

$$\text{clamp}_{[a,b]}(x) = \max\{a, \min(b, x)\}.$$

Unless stated otherwise, states are code-normalized and do not have declared physical units.

Neuron agent

With calcium C, tau phosphorylation T, and viability V, assignment order matters:

$$C_{t+1} = C_t + 0.05A_t\Delta t, \tag{1}$$
$$T_{t+1} = T_t + 0.02C_{t+1}\Delta t, \tag{2}$$
$$V_{t+1} = \max\{0, V_t - 0.1T_{t+1}\Delta t\}. \tag{3}$$

There is no random term. Only viability has a lower clamp. Defaults are $C_0 = 0.1$, $T_0 = 0$, and $V_0 = 1$. The 12 `GENE_MAP` values are model-assigned relative weights and are not transcriptomic measurements.

Astrocyte agent

Let R denote reactivity, Y cytokine level, G glutamate uptake, and W calcium-wave output:

$$R_{t+1} = \min\{1, R_t + 0.15(Y_t + 0.1A_t)\Delta t\}, \tag{4}$$
$$G_{t+1} = \max\{0.1, 0.8 - 0.5R_{t+1}\}, \tag{5}$$
$$W_{t+1} = 0.3R_{t+1}. \tag{6}$$

There is no random term and no lower clamp on reactivity.

Microglia agent

Let N denote NF-κB activity and $\epsilon_N \sim \mathcal{N}(0, (0.01\Delta t)^2)$:

$$N_{t+1} = \text{clamp}_{[0,1]}\left[N_t + (0.1A_t - 0.05N_t)\Delta t + \epsilon_N\right].$$

An independent $r \sim U(0, 1)$ redraws activation state according to Table S7a.

Table S7a. Microglial state probabilities

NF-κB interval	$P(M1)$	$P(M2)$	$P(M0)$
$N_{t+1} > 0.6$	0.75	0.20	0.05
$0.3 < N_{t+1} \leq 0.6$	0.30	0.40	0.30
$0.1 < N_{t+1} \leq 0.3$	0.10	0.20	0.70
$N_{t+1} \leq 0.1$	0	0.05	0.95

State-dependent raw phagocytosis is $0.1 + \epsilon_1$ for M1, $0.6 + \epsilon_2$ for M2, and $0.3 + \epsilon_0$ for M0, followed by clamping to $[0, 1]$. M1 outputs TNF-$\alpha = 0.8N$ and IL-1$\beta = 0.6N$; M2 outputs IL-10$= 0.3N$ and TGF-$\beta = 0.2N$.

Oligodendrocyte agent

Let M denote myelination capacity, I myelin integrity, P maturation progress, C calcium, and F TNF-α:

$$M_{t+1} = \text{clamp}_{[0,1]}(M_t - 0.05A_t\Delta t - \epsilon_M), \tag{7}$$
$$I_{t+1} = \text{clamp}_{[0,1]}(I_t - 0.08C_t\Delta t - \epsilon_I), \tag{8}$$
$$s_t = 1 - \min(1, 0.7F_t), \tag{9}$$
$$P_{t+1} = \text{clamp}_{[0,1]}[P_t + 0.02s_t\Delta t + \epsilon_P]. \tag{10}$$

The unidirectional transition is OPC to pre-OL at $P \geq 0.4$, then pre-OL to mature-OL at $P \geq 0.8$ on a later call.

Endothelial agent

Let J denote tight-junction strength, B BBB integrity, F TNF-α, L IL-10, and Q net amyloid-β transport:

$$J_{t+1} = \text{clamp}_{[0,1]}\{J_t + [0.01(1 - J_t) - 0.04A_t]\Delta t + \epsilon_J\}, \tag{11}$$

$$B_{t+1} = \text{clamp}_{[0,1]}\{B_t + [0.05(J_{t+1} - B_t) + 0.06L_t(1 - B_t) - 0.10F_t]\Delta t + \epsilon_B\}, \tag{12}$$

$$Q_{t+1} = \text{clamp}_{[-1,1]}[0.6B_{t+1} - 0.3(1 - B_{t+1}) + \epsilon_Q]. \tag{13}$$

The transport update replaces Q_t rather than forming an Euler increment. Positive values denote clearance and negative values denote influx.

Implementation caveats

Reported scalar states are rounded to four decimals by `get_state()`, while internal updates retain floating-point precision. Input validation differs by class. Coefficients remain heuristic/model-assigned unless a direct parameter-level source is encoded.

Supplementary analysis files

The accompanying archive contains patient-level virtual-trial visits, GEE terms, independent Monte Carlo trial summaries, APOE4 interaction output, ablation results, grid-convergence results, and SHA-256 provenance records under `paper/ijms_revision/`.
